# The Global Burden of Air Pollution on Mortality: Anenberg et al. respond

**DOI:** 10.1289/ehp.1002397R

**Published:** 2010-10

**Authors:** Susan C. Anenberg, J. Jason West, Larry W. Horowitz, Daniel Q. Tong

**Affiliations:** University of North Carolina at Chapel Hill, Chapel Hill, North Carolina, E-mail: jjwest@email.unc.edu; National Oceanic and Atmospheric Administration, Geophysical Fluid Dynamics Laboratory, Princeton, New Jersey; Science and Technology Corporation, Silver Spring, Maryland

We appreciate the comments by Rylance et al. stimulated by our analysis of the global burden of disease due to outdoor air pollution (Anenberg et al. 2010). We acknowledge that indoor air pollution is—and has long been recognized as—a significant burden on public health, particularly in developing countries where solid fuels are used extensively for cooking and heating (e.g., Smith 1987; Smith et al. 2004), but these comments on indoor air pollution do not affect our conclusions about the impacts of outdoor air pollution on global mortality.

In the 2004 World Health Organization (WHO) comparative risk assessment, Ezzati et al. (2004) estimated that indoor air pollution associated with household use of solid fuels is responsible for more premature mortalities than outdoor air pollution. Although our estimate of premature mortality due to outdoor air pollution is higher than the previous WHO estimate (Cohen et al. 2004), it should only be compared with indoor air pollution when methods for both risk factors are updated consistently, as in the forthcoming Global Burden of Diseases, Injuries, and Risk Factors Study (Institute for Health Metrics and Evaluation 2010).

We agree with Rylance et al. that the approach used by Pope et al. (2009) to integrate outdoor air pollution and cigarette smoking on a common scale would potentially also be useful for analyzing indoor air pollution. More broadly, additional research is needed to understand and differentiate indoor and outdoor exposures to multiple air pollutants and their ultimate effects on health in different parts of the world.

## References

[b1-ehp-118-a424b] Anenberg SC, Horowitz LW, Tong DQ, West JJ (2010). An estimate of the gobal burden of anthropogenic ozone and fine particulate matter on premature human mortality using atmospheric modeling. Environ Health Perspect.

[b2-ehp-118-a424b] Cohen AJ, Anderson HR, Ostro B, Pandey KD, Krzyzanowski M, Künzli N, Ezzati M, Lopez AD, Rodgers A, Murray CJL (2004). Urban air pollution. Comparative Quantification of Health Risks: Global and Regional Burden of Disease Attribution to Selected Major Risk Factors.

[b3-ehp-118-a424b] Ezzati M, Rodgers A, Lopez AD, Vander Hoorn S, Murray CJL (2004). Mortality and burden of disease attributable to individual risk factors.

[b4-ehp-118-a424b] Institute for Health Metrics and Evaluation (2010). Global Burden of Diseases Study.

[b5-ehp-118-a424b] Pope CA, Burnett RT, Krewski D, Jerrett M, Shi Y, Calle EE (2009). Cardiovascular mortality and exposure to airborne fine particulate matter and cigarette smoke: shape of the exposure-response relationship. Circulation.

[b6-ehp-118-a424b] Smith KR (1987). Biofuels, Air Pollution, and Health: a Global Review.

[b7-ehp-118-a424b] Smith KR, Mehta S, Maeusezahl-Feuz M, Ezzati M, Lopez AD, Rodgers A, Murray CJL (2004). Indoor air pollution from household use of solid fuels. Comparative Quantification of Health Risks: Global and Regional Burden of Disease Attribution to Selected Major Risk Factors.

